# Selective Microwave Zeroth-Order Resonator Sensor Aided by Machine Learning

**DOI:** 10.3390/s22145362

**Published:** 2022-07-18

**Authors:** Nazli Kazemi, Nastaran Gholizadeh, Petr Musilek

**Affiliations:** 1 Electrical and Computer Engineering, University of Alberta, Edmonton, AB T6G 1H9, Canada; nazli@ualberta.ca (N.K.); nastaran@ualberta.ca (N.G.); 2 Applied Cybernetics, University of Hradec Králové, 500 03 Hradec Králové, Czech Republic

**Keywords:** microwave sensor, selectivity, resonators, machine learning, generative adversarial network

## Abstract

Microwave sensors are principally sensitive to effective permittivity, and hence not selective to a specific material under test (MUT). In this work, a highly compact microwave planar sensor based on zeroth-order resonance is designed to operate at three distant frequencies of 3.5, 4.3, and 5 GHz, with the size of only $\lambda_{g-min}/8$ per resonator. This resonator is deployed to characterize liquid mixtures with one desired MUT (here water) combined with an interfering material (e.g., methanol, ethanol, or acetone) with various concentrations (0%:10%:100%). To achieve a sensor with selectivity to water, a convolutional neural network (CNN) is used to recognize different concentrations of water regardless of the host medium. To obtain a high accuracy of this classification, Style-GAN is utilized to generate a reliable sensor response for concentrations between water and the host medium (methanol, ethanol, and acetone). A high accuracy of 90.7% is achieved using CNN for selectively discriminating water concentrations.

## 1. Introduction

Microwave planar sensors have recently spread in a wide range of applications including material characterization [1,2], the oil and sand industry [3,4,5], the biomedical field [6,7,8], and many others [9,10]. The main motivation behind the growing interest in these sensors is a combination of their intriguing features (such as low-cost mass production, simple design, high sensitivity) and, more importantly, non-contact mode of sensing. The latter allows for the interaction of materials in the proximity of the sensor to be interrogated without direct contact. This enables the operation of the sensor in harsh environments and inaccessible areas. This eventually prolongs the sensor life as the maintenance cost significantly decreases. However, this contact-less sensing scheme becomes problematic when the material under test (MUT) resides within an unknown background material. Similarly, when an interfering medium/material is present in the sensing range of a microwave planar sensor, the measurement results are affected. The microwave sensing principle in resonator-based sensors is attributed to the effective dielectric load on the resonator. It is clear that the mixture of several liquids modifies the dielectric properties, which can be quantified using the Maxwell–Garnett equation [11], compared with that of only a single MUT. In addition, unintended changes in the environment that impact the effective dielectric properties (e.g., relative humidity [12] or temperature [13]) may also lead to inaccuracies in the sensing outcome. As a result, planar sensing has complexities that need to be resolved in the microwave regime.

This article elaborates on a common issue among others of microwave sensors that are sensitivity to the effective medium rather than a specific MUT. It then proposes a technique to overcome this issue using machine learning. In a complex matrix consisting of two MUTs, one can determine the contribution of each material in the solution using a continuous regression model of the sensor response, e.g., using resonance frequency. The reason for the choice of resonance frequency is its robustness to the environmental thermal noise in comparison to the resonance amplitude. This regression is easily computed when the background medium is known. However, when the desired MUT is mixed with another, unknown liquid, it becomes erroneous to identify the level of desired MUT. In this work, we propose a multi-resonance sensor that is empowered with a machine learning algorithm to quantify the level of selected MUT in a binary mixture regardless of the host medium. This resonance-based process [14,15,16,17] yields a sensor that is not only sensitive, but also selective to only a single MUT, a characteristic that is not inherent to microwave sensors. The methodology used to differentiate various concentrations of the desired MUT (from 0→100%) is discretized into 11 groups using a convolutional neural network (CNN). Since CNN typically takes an image as the input, a novel approach is employed to convert the sensor measurement into an image, known as a heat map, suitable for the subsequent machine learning blocks. Deep learning has been used in various areas including biomedical [18,19,20,21] and agricultural applications [22,23], wherein self-feature extraction is exploited and learned through CNN [24,25].

It should be noted that using deep learning CNN as the classifier requires a large dataset for training. Using n extensive training set, CNN outperforms other methods such as ImageNet [26]. However, this process is intrinsically time consuming due to the need to individually label the measured samples. This results in a limited number of available training data points and, subsequently, in an overfitted trained model. At the same time, hand-crafted measurements are prone to error, especially for small concentrations of the desired MUT. To address the challenges associated with the use of CNN for material characterization, this paper introduces a technique to generate a large number of data points with high accuracy using a generative adversarial network (GAN) [27,28] called Style-GAN. It has been shown that GANs can be used for image generation [29], audio generation [30], and language processing [31]. However, the specifics of image generation are not sufficiently determined to allow controlled manipulation of the generated image. In contrast, Style-GAN as a variant of GANs, can generate realistic images because of the presence of latent vectors, which allow direct manipulation of the generated images. In this article, Style-GAN is used to generate a large number of suitable sensor responses for all intermediate concentrations between the selected MUT (here water) and undesired background material. This approach generates images with high accuracy, suitable for a CNN-based classification algorithm. In turn, it facilitates the effective evaluation of the sensor response for an arbitrary binary mixture.

The are two main novel contributions of the proposed approach as follows. First, the generation of a large number of synthetic sensor responses is of utmost importance specifically when measurement requires huge resources for high accuracy. This requirement is successfully met using Style-GAN (i.e., data augmentation). Second, the level of selected MUT is assessed by classifying the sensor response considering not just a single host medium, but several different media. Enabling this selectivity in sensing for a microwave sensor is conducted using a CNN that is trained to realize a selective microwave sensor. All in all, a significantly compact design is developed to be sensitive to only a single material of interest.

## 2. Sensor Design and Analysis

**Figure 1:**
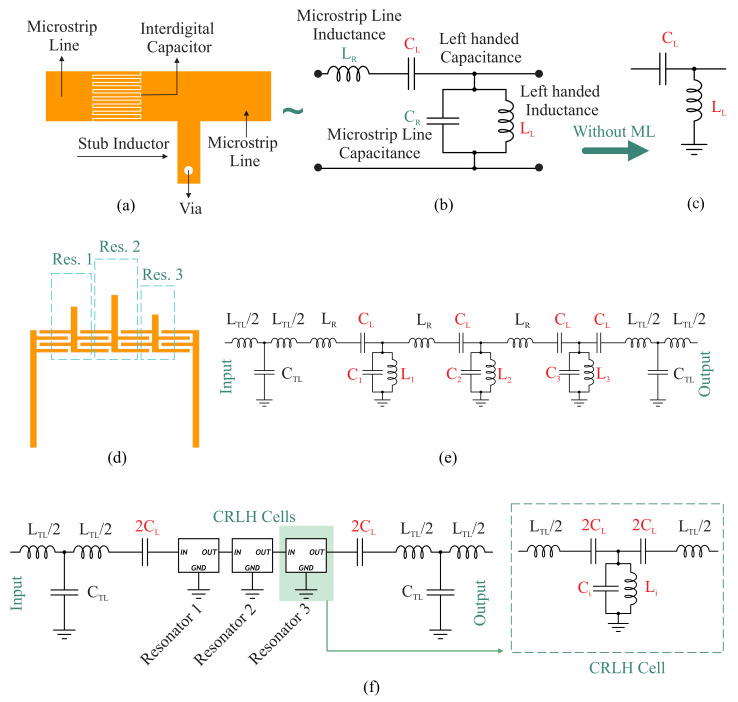
Composite right-/left-hand transmission line unit cell in (**a**) layout and (**b**) equivalent circuit model with series and shunt resonators. (**c**) A typical left-handed transmission line without considering the right-handed transmission line components. (**d**) Layout of the proposed sensor with three adjacent resonators sharing similar capacitors and different stubs. (**e**) Equivalent circuit model of the proposed multi-resonator-based sensor. (**f**) Simplified circuit model with blocks of CRLH cells.

The unique properties of left-handed metamaterials introduced sophisticated devices with novel electromagnetic propagation properties. Reported structures include a resonant approach using SRR and metal wire [32] and a non-resonant one using an interdigital capacitor (IDC) and stubs [33]. The use of SRR and wire in the microwave regime is cumbersome due to the size limitations and narrow bandwidth. The transmission line approach, however, results in regions of operation for a positive, negative, or even zero phase constant (β). A typical series IDC and parallel stub, shown in Figure 1a, is essentially comprised of a series capacitor $C_{L}$ and a parallel inductor $L_{L}$. In addition, the inductance of the series IDC and the capacitance between the stub and the ground, known as $L_{R}$ and $C_{R}$, respectively, are used to comprehensively represent the equivalent circuit model of the unit cell. This combination leads to a composite right-/left-handed transmission line (CRLH TL). It can be designed for balanced resonances between series $\omega_{se}$ and shunt $\omega_{sh}$ tanks as follows:

(1) $$\omega_{se}=\frac{1}{\sqrt{L_{R}C_{L}}},$$

(2) $$\omega_{sh}=\frac{1}{\sqrt{L_{L}C_{R}}}.$$

The propagation constant $\beta_{CRLH}$ can be computed according to:

(3) $$\left|\beta_{CRLH}\right|=\sqrt{\omega^{2}L_{R}C_{R}+\frac{1}{\omega^{2}L_{R}C_{L}}-\left(\frac{L_{R}}{L_{L}}+\frac{C_{R}}{C_{L}}\right)}.$$

In the case of a zero propagation constant (β=0) from (3), an infinite wavelength is supported.

The resonances of an open-ended CRLH TL occur when the propagation constant of each resonance $\beta_{n}$ is defined as:

(4) $$\beta_{n}=\frac{n\pi }{l},\;\;\;\mathrm{with}\;\;\;n=0,\pm 1,\pm 2,\cdots ,\pm \left(N-1\right),$$

where l,n, and *N* represent the length of the resonator, mode number, and number of unit cells, respectively. It is apparent that with β=0, the wavelength $\lambda_{g}=2\pi /\beta_{n}$ becomes infinite and the resonance frequency is no longer tied to the sensor size. In comparison, the resonator size for a conventional open-ended resonator is half-wavelength. This feature of zeroth-order resonators allows for significant miniaturization, which is the motivation for this choice of resonator, especially when three resonators are used for a multi-resonance application. Since the material permittivity changes across frequency, it is recommended to study the material behavior in a broadband or multi-frequency span. In this design, three frequencies are chosen to cover a relatively large frequency span. In order to implement resonators associated with this frequency, the series capacitance and shunt inductance of a zero-order resonator can be arbitrarily chosen. To have a compact and consistent sensitive region for the MUT, the architecture of the IDC is shared among all resonators, while the length of the shunt inductors determines the resonance frequencies; therefore, one can obtain the resonance frequency of interest for each resonator by tuning the length of the corresponding shunt inductor. It is noteworthy to mention that the placement order of resonators affects the amplitude of transmission specifically for the lowest resonance. Considering this fact, the shunt inductor related to the lowest resonance frequency is located in the middle followed by feedback from the simulation results.

### 2.1. CRLH Analysis

The CRLH unit cell for the zeroth-order resonator is depicted in Figure 1a, which is essentially comprised of a series IDC and a stub shorted with a via. This is equivalent to a composite of series and parallel resonators, where the engineered series IDC fingers contribute to both capacitance and inductance. A similar assumption holds true for the shunt stub, which possesses capacitive features between the stub and the ground, while the stub acts as an inductor due to its short length (<$\lambda_{g}/4$). The pure left-handed TL representation is given in Figure 1c as well, where only the main contributing components, i.e., the series capacitance $C_{L}$ and shunt inductance $L_{L}$, are presented. However, the presence of the underlying copper traces acting as $C_{L}$ and $L_{L}$ is significant enough to consider the regular representation of a right-handed TL as well.

The proposed resonator sensor is depicted in Figure 1d, where three zeroth-order resonators are cascaded with shared IDCs and separate shunt inductors. This sensor is comprised of three distinct resonance frequencies differentiated by specific stub lengths. The equivalent circuit model of the whole multi-resonator sensor is shown in Figure 1e, which is indeed a combination of three CRLH TLs in Figure 1b. Each capacitor is shared between two resonators; hence, in the corresponding circuit model given in Figure 1f, each series $C_{L}$ is doubled to illustrate each CRLH TL unit cell with a symmetrical representation. The regular TL circuit model denoting the input and output of the sensor is given as a symmetric series inductance ($L_{TL}/2$) and shunt capacitance ($C_{TL}$).

The relationship between the theoretical parameters ($S_{21}$ and resonance frequency) and dielectric properties of the MUT can be derived through the following analysis: Accurate modeling of the sensor response is conducted through ABCD matrix analysis. Since the individual components of the sensor are cascaded, the whole design is divided into three main subsections including two transmission lines in the input and output ($T_{I/O}$), three individual CRLH resonators ($C_{i}$, *i* = 1, 2, 3), and two series capacitances ($2C_{L}$), as shown in Figure 1f.

#### 2.1.1. Input/Output Transmission Line

The two transmission lines at the input and output of the sensor with the length of $l_{I/O}$, propagation constant β, and characterization impedance $Z_{0}=1/Y_{0}$ are modeled as follows:

(5) $$T_{I/O}=\left[\begin{array}{cc} cos(\beta l_{I/O}) & jZ_{0}sin\left(\beta l_{I/O}\right)\\ jY_{0}sin\left(\beta l_{I/O}\right) & cos(\beta l_{I/O}) \end{array}\right]$$

#### 2.1.2. Connecting Capacitances

The two marginal capacitances as shown in Figure 1f are modeled as follows:

(6) $$T_{2C_{L}}=\left[\begin{array}{cc} 1 & \frac{1}{j\omega 2C_{L}}\\ 0 & 1 \end{array}\right]$$

where $2C_{L}$ is impacted by the MUT ($\varepsilon_{MUT}$) and is modified as follows:

(7) $$2C_{L}\;\;\;⟹\;\;\;2C_{L}^{\prime }=2C_{L}\frac{\varepsilon_{r}+\varepsilon_{MUT}}{\varepsilon_{r}+1}$$

#### 2.1.3. CRLH Model

Each individual resonator is modeled with a cascade of capacitors and inductors, as shown in Figure 1f, which allows for matrix multiplication to determine the overall transfer function of each CRLH unit cell.

(8) $$T_{CRLH_{i}}=\left[\begin{array}{cc} 1 & j\omega L_{TL}/2\\ 0 & 1 \end{array}\right]\left[\begin{array}{cc} 1 & \frac{1}{2j\omega C_{L}^{\prime }}\\ 0 & 1 \end{array}\right]\left[\begin{array}{cc} 1 & 0\\ j\omega C_{i} & 1 \end{array}\right]\left[\begin{array}{cc} 1 & 0\\ \frac{1}{j\omega L_{i}} & 1 \end{array}\right]\left[\begin{array}{cc} 1 & \frac{1}{2j\omega C_{L}^{\prime }}\\ 0 & 1 \end{array}\right]\left[\begin{array}{cc} 1 & j\omega L_{TL}/2\\ 0 & 1 \end{array}\right]$$

which can be summarized as follows:

(9) $$T_{CRLH_{i}}=\left[\begin{array}{cc} A_{i} & B_{i}\\ C_{i} & D_{i} \end{array}\right]$$

The total transfer function is obtained by cascading all elements as follows:

(10) $$T_{I/O}\times T_{2C_{L}^{\prime }}\times T_{CRLH_{1}}\times T_{CRLH_{2}}\times T_{CRLH_{3}}\times T_{2C_{L}^{\prime }}\times T_{I/O}=\left[\begin{array}{cc} A & B\\ C & D \end{array}\right]$$

Therefore, scattering parameter $S_{21}$ can be obtained with the following conversion equation:

(11) $$S_{21}=\frac{2}{A+B/Z_{0}+CZ_{0}+D}$$

The corresponding resonance frequency of each resonator can be obtained through the equation when $\beta_{CRLH}$ = 0, as shown in Equation (2).

### 2.2. Sensor Topology and Field Analysis

**Figure 2:**
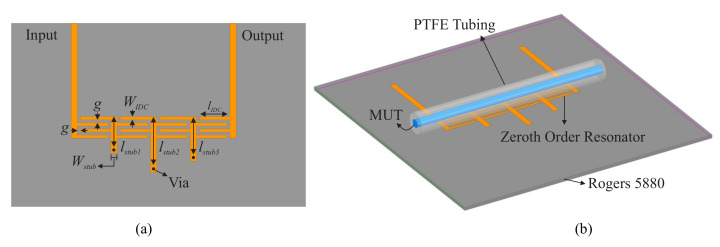
(**a**) Top-view of the proposed multi-resonator sensor. (**b**) Perspective of the sensor with the fluidic channel mounted on the top.

A detailed top-view of the proposed sensor is shown in Figure 2a with the dimensions also given in Table 1. The structure is exposed to the MUT within a fluidic channel model, shown in Figure 2b, where the tubing is designed to cover the whole IDC section to exploit the fringing fields. Extending the tube to cover all IDCs engages all resonators and affects their resonance profiles simultaneously. This way, a multi-resonant sensor is structured, which is utilized to enable the characterization of a material over a wide range of frequencies.

The multi-resonator sensor was simulated in Ansys Electronics (HFSS) considering the bare resonator without any material/tube on it. HFSS uses a numerical method called the finite element method. Finite elements refer to smaller subsections that the desired structure is divided into. A solution for the fields (electric/magnetic) within the entire finite elements (mesh) is achieved when Maxwell’s equations are satisfied across inter-element boundaries. Once the field solution is available, the scattering parameters of the design are obtained. Mathematically, considering the boundary conditions and excitations, HFSS solves for the electric field *E* as follows:

(12) $$\nabla \times \left(\frac{1}{\mu_{r}}\nabla \times E\right)-K_{0}^{2}\varepsilon_{r}E=0$$

where $\mu_{r}=\frac{\mu }{\mu_{0}}$, $\varepsilon_{r}=\frac{\varepsilon }{\varepsilon_{0}}$, $K_{0}^{2}=\omega^{2}\varepsilon_{0}\mu_{0}=\frac{\omega^{2}}{c^{2}}$, and for the magnetic field H as follows:

(13) $$H=\frac{1}{\omega \mu }\nabla \times E$$

**Figure 3:**
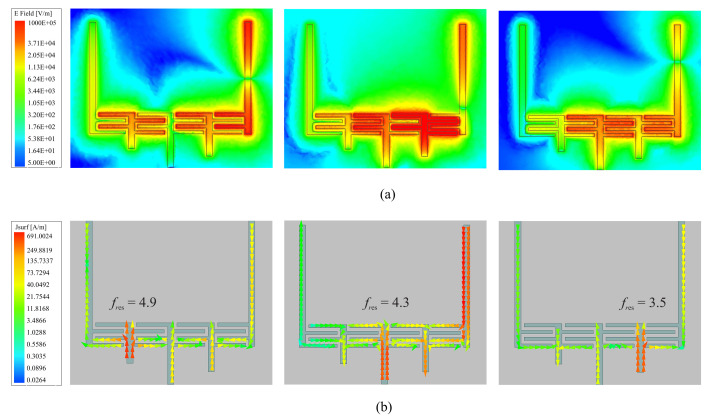
Sensor field analysis in HFSS with (**a**) magnitude of electric field $\left|E\right|$ and (**b**) magnitude of surface current $\left|J_{surf}\right|$.

**Figure 4:**
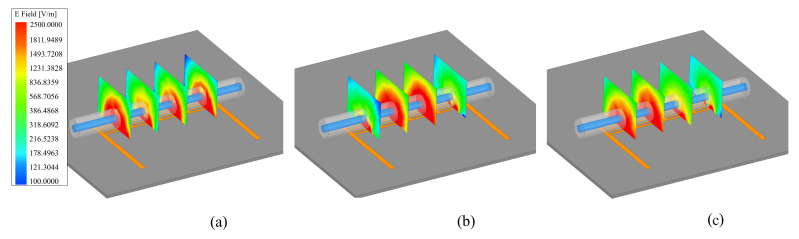
Magnitude of electric field $\left|E\right|$ with the filled tube on the sensor at all resonances of (**a**) 4.9 GHz, (**b**) 4.3 GHz, and (**c**) 3.5 GHz.

The magnitude of electric fields, shown in Figure 3a, also depicts the location of highly concentrated regions at a specific frequency that corresponds to the resonance of each resonator. For one, the shortest stub is associated with the highest resonance frequency, as shown in Figure 3a (left), with high electric fields concentrated on the left-most resonator. Please note that, in these simulations, the input port is located at the right side. Following the same inverse proportionality between the resonance frequency and the stub length, the next two resonances are also represented in the middle/right figures for the middle and lowest resonance frequencies, respectively. All resonances at 3.5, 4.3, and 4.9 GHz are also correlated with the corresponding magnitude of surface currents on the microstrip traces, as shown in Figure 3b. It is evident that the surface currents are maximally excited at each resonator with the corresponding resonance frequency, while affecting other resonators to a minimal degree. In addition, to demonstrate the depth of electric fields into the free space, $\left|E\right|$ is depicted in Figure 4a–c. It is also clear that with the presence of tube filled with an analyte, here water, the surrounding volume around a resonator also becomes deeply interrogated compared with the adjacent locations. This also reaffirms that the resonators are able to interact with an MUT deep inside the fluidic tube.

### 2.3. Sensor Response in Simulation

**Figure 5:**
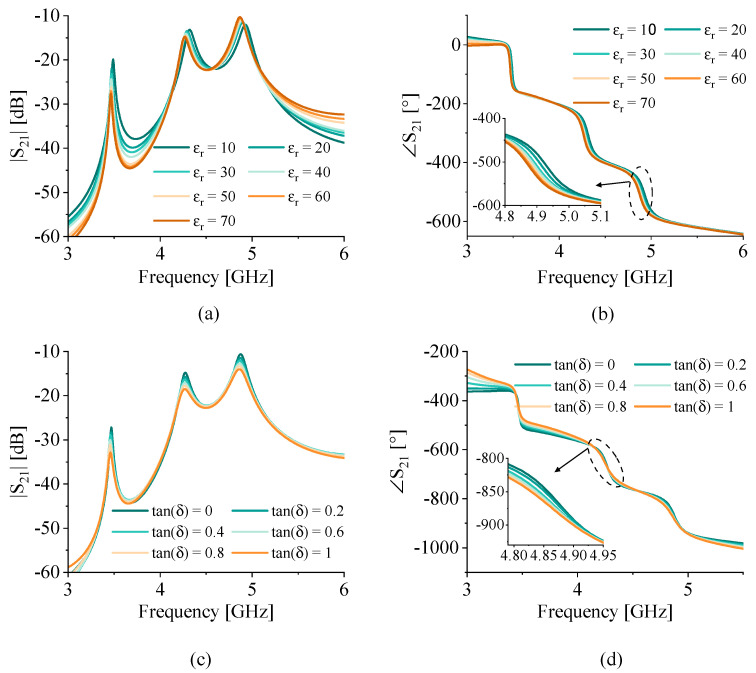
Sensor performance for a filled tube on the sensor. The effect of variable permittivity and tan(δ)=0 on (**a**) $\left|S_{21}\right|$ and (**b**) $∡S_{21}$ and loss tangent variation with $\varepsilon_{r}=70$ on (**c**) $\left|S_{21}\right|$ and (**d**) $∡S_{21}$.

In this section, the fluidic tube holding the desired MUT is placed on the IDCs and the sensor response is simulated for various dielectric properties of the MUT. This performance analysis involves both permittivity $\varepsilon_{r}$ and the loss tangent tan(δ). First, the permittivity of the MUT inside the tubing is varied from 10 to 70 with increments of 10, while the loss tangent is kept constant at tan(δ)=0. This allows interpreting the results and their sensitivity to only the real permittivity variation. The magnitude and phase of transmission profiles $S_{21}$ are depicted in Figure 5a,b, respectively. It is clear that the three resonances are separate enough from each other to avoid significant mutual loading. The resonance frequencies decrease while the permittivity increases, which is apparent in both the magnitude and phase profiles. In another exercise, shown in Figure 5c,d, the effect of the loss tangent was studied. It is shown that increasing the value of tan(δ) from 0 up to 1 with an increment of 0.2 reduces the sharpness of the resonances and their amplitude. This lowered amplitude of resonance translates to the lowered slope of the $∡S_{21}$ with shallower curves.

It should be noted that the sensor response depends on the equivalent dielectric properties of the MUT over a wide range of frequencies. In this simulation, however, only a fixed value is used for both $\left|S_{21}\right|$ and $∡S_{21}$, which is an averaged value at 4.5 GHz. This method not only reduces the simulation time, but also holds high accuracy with respect to the trends in the sensor response.

## 3. Measurement Results and Discussion

### 3.1. Measurement Setup

**Figure 6:**
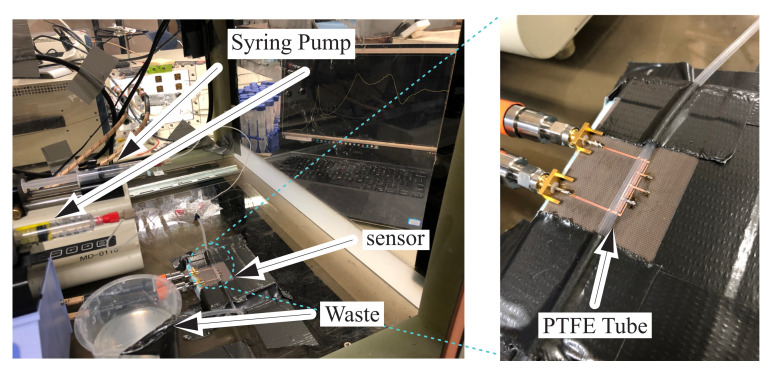
Measurement setup with two syringe pumps and the fluidic PTFE tube mounted on the sensor.

The measurement setup includes a S5065 copper mountain vector network analyzer (VNA), which is connected to the sensor with two STABILITY Microwave/RF (SC-N) cables as shown in Figure 6. Two syringe pumps, Pump 11 Elite (gray) and Cole-Parmer single syringe infusion pump (CAT No.78-9100C) (blue), were used to inject the MUT through a polytetrafluoroethylene (PTFE) fluidic channel. A plastic container was used to collect the waste material from the fluidic channel. The whole system resides inside a cubic chamber, which is covered by a foam sheet broadband microwave absorber (ECCOSORB-AN79) to protect the sensing environment from microwave inferences.

### 3.2. Results and Discussion

**Figure 7:**
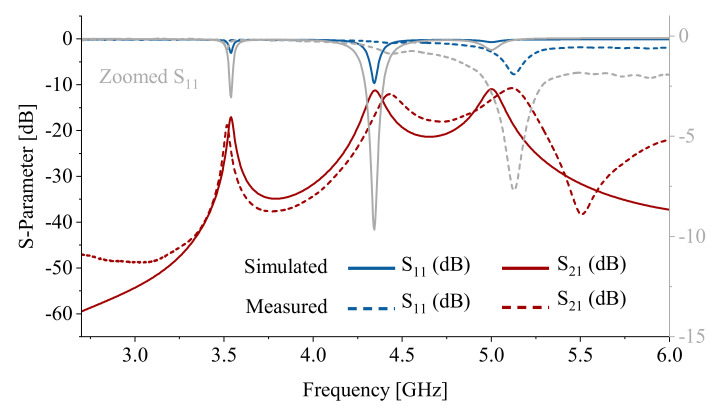
Comparison between measured (solid) and simulated (dashed) scattering parameters in magnitude (blue) and phase (red).

The proposed multi-resonator sensor was fabricated on the Rogers 5880 substrate with the parameters given in Table 1. The sensor was etched with ammonium persulfate dissolved in water. Using Copper Mountain S5065, transmission/reflection parameters in both magnitude (red) and phase (blue) were measured, as shown in Figure 7. A polytetrafluoroethylene (PTFE) fluidic channel with outer diameter $={1/8}^{\prime \prime }$ and inner diameter $={1/16}^{\prime \prime }$ was mounted on the sensor surface, allowing the MUT to flow through. An initial measurement response of the sensor without any liquid inside is shown in Figure 7. The measured resonance profile matches with the simulation result and verifies the three resonances in both the frequency and magnitude of resonance for either transmission $S_{21}$ or reflection $S_{11}$ (zoomed in with grey color) scattering parameters. Some minor discrepancies go back to the fabrication errors for the in-house etching process, as well as to the implementation of vias.

In this work, several binary mixtures were prepared with water in a secondary liquid, here including methanol, ethanol, and acetone. In order to achieve different mixtures with various concentrations of water, two syringe pumps were used. The syringe pumps were tuned to allow different flow rates for the constituting liquids of a mixture. As a result of a proper flow rate for the syringe holding water, concentrated solutions with increments of 10% were obtained.

Since the microwave sensor is tightly linked with the effective dielectric constant of the solution, their effective permittivity was calculated through the Cole–Cole model with parameters taken from [34,35,36] as follows:

(14) $$\varepsilon =\varepsilon_{\infty }+\frac{\left(\varepsilon_{0}-\varepsilon_{\infty }\right)}{1+{\left(j\omega \tau \right)}^{1-\alpha }},$$

where τ is the relaxation time constant, α is an empirical parameter, and $\varepsilon_{0}$ and $\varepsilon_{\infty }$ are the low-frequency and high-frequency dielectric constant, respectively.

The propagation of electromagnetic waves inside a medium is tightly linked to the constituent parameters of the medium including the permittivity ($\varepsilon_{r}$) and loss tangent (tan(δ)). Considering different concentrations of water when the permittivity increases at a given frequency, the wave speed that is determined by $v=\frac{1}{\sqrt{\mu \varepsilon }}$ reduces. Similarly, the wavelength decreases according to → ($\lambda =\frac{v}{f}$). On the other hand, propagation in a lossy medium with a non-zero loss tangent affects the wave amplitude by a factor of $e^{\alpha z}$, where $\alpha =\omega \sqrt{\frac{\mu \varepsilon }{2}\left[\sqrt{1+tan{\left(\delta \right)}^{2}}-1\right]}$.

**Figure 8:**
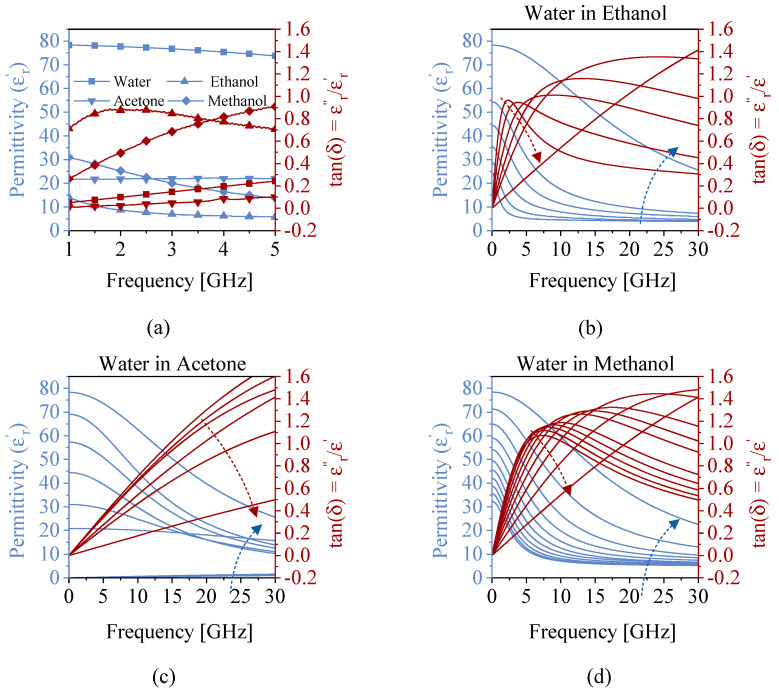
Dielectric properties of the bulk medium including water, methanol, ethanol, and acetone (**a**) and of concentrated solutions of water in ethanol (**b**), acetone (**c**), and methanol (**d**). Please note that the arrows demonstrate the increase in the water concentrations from 0−100%.

**Figure 9:**
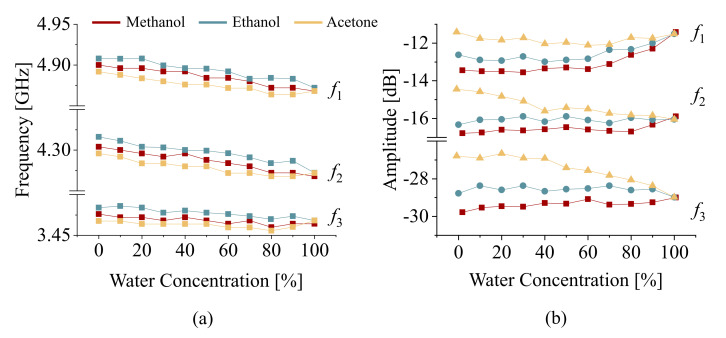
Measured sensor response for various concentrations of water in methanol/ethanol/acetone in both (**a**) resonance frequency and (**b**) amplitude of resonance.

**Figure 10:**
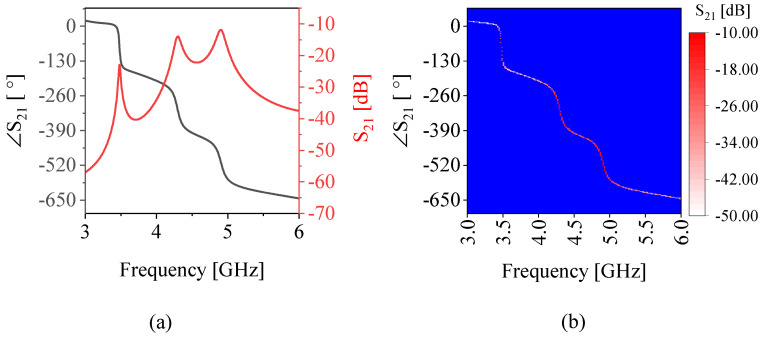
(**a**) Magnitude and phase of resonator sensor response. (**b**) Corresponding $S_{21}$ phase vs. frequency with the color intensity determined by the $|S_{21}|$ level.

Figure 8a depicts the bulk medium permittivity values at the wide range of frequencies covering the span of interest. Subsequently, the binary mixtures of water in methanol, ethanol, and acetone are also computed and plotted in Figure 8b–d. It is apparent that the relative permittivity decreases over frequency for all solutions; however, the trend of the loss tangent is not predictable. With this in mind, various instances at a given frequency exist wherein the relative permittivity values overlap, which results in a similar performance for the frequency of operation in all three resonances of the proposed sensor. This leads to an indecisive conclusion on the nature of the ingredients since more than one candidate can be found that result in the same effective permittivity. This suggests that the effect of the background medium is considerable on the sensor response. The impact of background material is more elaborated when the concentrated solutions of water in methanol/ethanol/acetone are tested within a PTFE tubing. Measurements were recorded using LabView with time interval of 5 s, leading to a total of 50 measured sensor responses per intermediate concentration. The corresponding resonance frequencies are down-shifted in Figure 9 as a result of increased water content (equivalent to increased effective permittivity), as shown in Figure 8b–d. The trend is more significant for ethanol compared to the other MUT due to its lower permittivity at the frequency of interest compared to methanol or acetone. It can also be inferred that the measured sensor parameters of resonance, such as frequency and amplitude, do not independently suggest the nature of the ingredients in a binary mixture. In other words, a single resonance frequency value can be attributed to more than one MUT. Therefore, the feature of the microwave sensor that is sensitive to the effective permittivity leaves the sensor non-selective to the MUT. In order to resolve this issue, in this work, a CNN-based classifier was used to distinguish different mixtures using feature extraction. Since CNNs depend on image-based data, the measured sensor response needs to be converted to a graphical representation. To address this issue, a novel representation was obtained based on the amplitude and phase of transmission $S_{21}$, as shown in Figure 10a. To obtain an image from these results, the phase diagram vs. frequency was kept as the baseline and the magnitude of transmission was used as the color intensity between white (low $|S_{21}|$) and red (high $|S_{21}|$), which is called a heat map in this work and shown in Figure 10b. This way, the results are completely conveyed in a unified format from which the constituent parameters can also be recovered. An image representation of 256×256×3 is fed to the network as the input data for a given transmission profile.

### 3.3. Style-GAN

Different concentrations of water in MUTs need to be meticulously prepared with proper flow rate adjustment in the syringes. Generating a high number of input data points for CNN requires time-consuming and laborious hands-on work, which is prone to error in sample preparation/testing. This is the main motivation to use an automated response generation for the sensor in its linear mode of operation. Please note that the fluidic channel cross-section is small and loads the sensor minimally, thus helping to exploit the linear mode of the sensor. In this section, a machine learning algorithm was employed to generate images with characteristics derived from two sources linearly using Style-GAN according to the linear mode of the sensor. Style interpolation incorporates two latent source imagesof $\omega_{1}$ and $\omega_{2}$ to generate a new style $\omega =\lambda \;\cdot \;\omega_{1}+\left(1-\lambda \right)\;\cdot \;\omega_{2}$, where λ stands for a strength parameter towards a class direction. This method was introduced by Keras et al. of Nvidia Research in 2018 [37]. Since then, Style-GAN generator has been applied in various applications including the generation of human faces and medical image synthesis [38,39]. Inspired by its wide-ranging application potential, this method was also used in the proposed microwave sensor.

**Figure 11:**
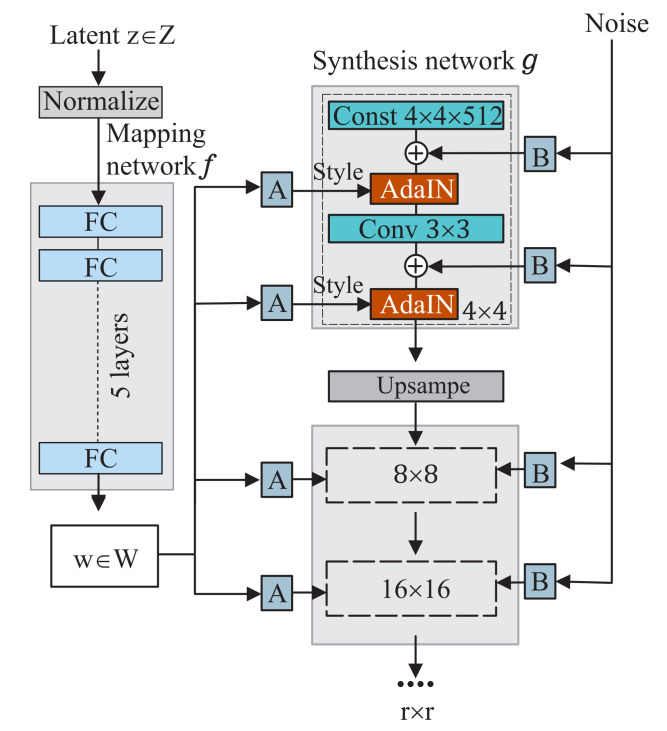
The style-based generator architecture.

A Style-GAN is characterized by two inputs of noise vectors and a randomized latent vector. The noise generated from a normal distribution creates stochastic variations, and the randomized vector sampled from a normal distribution determines global features in an image. This combination of latent vector and noise results in a variety of generated images. The architecture of Style-GAN is shown in Figure 11, which is composed of a mapping network *f* and a synthesis network *g*. The mapping network *f* is a multilayer perceptron with a latent code z∈Z, where *z* is a 512×1 vector sampled from a normal distribution N(0,1). This network outputs a style vector w∈W with the shape of 512×1. The purpose of the mapping network is to enable *w* to learn the distribution of the training set. The synthesis network *g* uses noise and style vector *w* to generate new images. This network is comprised of 14 (or 16–18) layers with seven (or 8–9) blocks for the image resolution of $256^{2}$ (or 512${}^{2}$–1024${}^{2}$). Each block consists of six operations: upscaling (1×), noise addition (2×), AdaIN (2×), and 3×3 convolution (1×). The operations of all blocks are similar except the first one, where a constant tensor is initialized. In each block, vector *w* and noise are used as the inputs. The affine transformation, shown with 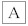 in Figure 11, converts vector *w* into the style mean μ and the style variance σ. An adaptive instance normalization uses the statistical information of μ and σ to pass the properties of the adapted style to the next one as follows:

(15) $$AdaIN\left(x_{i},y\right)=y_{s,i}\;\cdot \;\frac{x_{i}-\mu x_{i}}{\sigma (x_{i})}+y_{b,i},$$

where $y_{s,i},\;y_{b,i}$ stands for the standard deviation and mean of the adapted style, respectively; $x_{i}$ is the feature tensor passed from the previous operations.

The scaling per channel, shown with 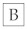 in Figure 11, fits the shape of the noise into the propagating tensor and sums them. A final RGB convolution is applied to the output, which transforms the tensor into a desired image of sensor response.

In this work, Style-GAN Encoder was used [40] to modify the high-level attributes. Mapping network *f* transforms *Z* drawn from normal distribution N(0,1) to *W* to enable high-end attribute encoding. The new images generated by style interpolation utilize the linear relationship $\omega =\lambda \;\cdot \;\omega_{1}+\left(1-\lambda \right)\;\cdot \;\omega_{2}$, which results in a smooth transition between the source and target images.

**Figure 12:**
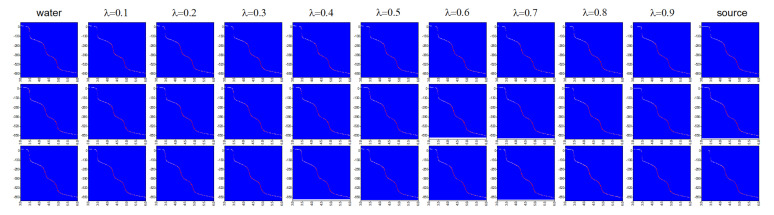
Interpolation between heat maps of the source materials of water ($S_{1}$) and the next source $S_{2}$ from methanol, ethanol, and acetone in Rows 1, 2, and 3, respectively.

The source materials of $S_{1}$ (water) and $S_{2}$ (methanol/ethanol/acetone) are measured with LabView every 5 s to arrive at 1000 measurements per sample. These sensor responses are converted into heat maps in order to train StyleGAN. The generated images between each set of $S_{1}$ and $S_{2}$ are represented in Figure 12 with λ factors between 0.1 and 0.9 with increments of 0.1, which represent increases of the concentration by 10%. This graph represents the sensor responses for each concentration assuming there is a linear variation in the sensor response, which is inferred from Figure 9a. Desired concentrations of water in all above-mentioned materials with 10% increments are also measured with the sensor topology shown in Figure 6. These measured responses are used as true values for comparison with the generated responses by Style-GAN using the following metrics.

The similarity between two images is measured with the structural similarity index (SSIM) for each subimage with a side length of 11 as follows:

(16) $$\mathrm{SSIM}\left(x,y\right)=\frac{\left(2\mu_{x}\mu_{y}+c_{1}\right)\left(2\sigma_{xy}+c_{2}\right)}{\left(\mu_{x}^{2}+\mu_{y}^{2}+c_{1}\right)\left(\sigma_{x}^{2}+\sigma_{x}^{2}+c_{2}\right)},$$

where $\mu_{x}$ and $\mu_{y}$ are the average pixel intensity of the subimages x and y, respectively. The standard deviations of the subimages x and y are, respectively, $\sigma_{x}$ and $\sigma_{y}$, and the covariance of the two subimages is $\sigma_{xy}$. In addition, the peak-to-signal ratio (PSNR) is used for quality assessments as follows:

(17) $$\mathrm{PSNR}=10log_{10}\left(\frac{{\mathrm{MAX}}_{i}^{2}}{\mathrm{MSE}}\right),$$

where MAX is the highest scale value of the pixels and the mean of absolute error (MSE) is defined as follows:

(18) $$\mathrm{MSE}=\frac{1}{MNO}\sum_{x=1}^{M}\sum_{y=1}^{N}\sum_{z=1}^{O}{\left(Y_{\left(x,y,z\right)}-{\hat{Y}}_{\left(x,y,z\right)}\right)}^{2}$$

Finally, to evaluate the performance of Style-GAN, the Frechet inception distance (FID) is used.

**Figure 13:**
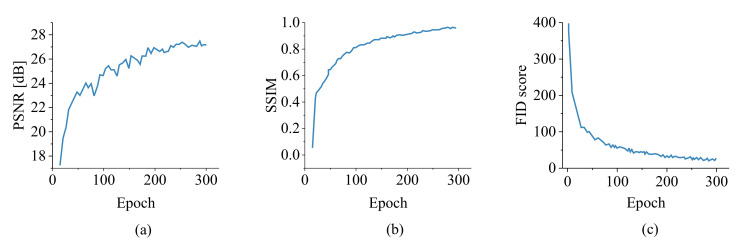
Image generation metrics using Style-GAN given as (**a**) PSNR, (**b**) SSIM, and (**c**) FID score.

These metrics are computed and shown in Figure 13a–c versus the number of epochs. Style-GAN converges at 300 epochs with PSNR=27.4dB, SSIM=0.859, and FID=21.6. The accuracy of the generated images was verified, which ensures that the generated images can be used as the input dataset to train CNN classification.

### 3.4. Classification Using CNN

The images generated using Style-GAN comprise the input set to train the classifier for different concentrations of water in various host media. This way, image representations of measured sensor responses are mapped to the corresponding concentrations of water. This model has two components: feature extraction in the front end (convolutional and pooling layers) and predictive classifier in the back end. The CNN model was evaluated for various combinations of parameters including the number of layers (1, 2, 3), optimizers (Adam, Stochastic gradient decent), learning rates (0.001, 0.01, 0.1), and loss functions (binary cross-entropy, categorical cross-entropy).

**Figure 14:**
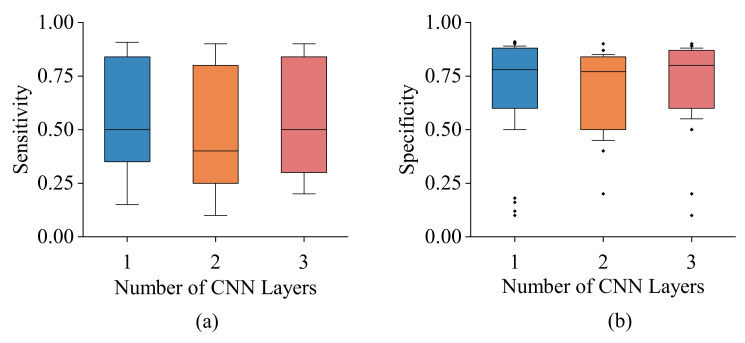
(**a**) Sensitivity and (**b**) specificity analysis for CNNs with different numbers of layers and hyperparameters including optimizers, learning rates, and loss functions. In the box plots the central line defines the median. The lower and upper hinges of the boxes correspond to the 25th and 75th percentiles, and the whiskers are the minimum and maximum values. Outliers that differ significantly from the rest of the dataset are plotted as individual points (diamond) beyond the whiskers on the box-plot.

Regarding the optimization of our model, our dataset has a specific format, magnitude, and phase of a frequency-based parameter, which is significantly different from well-known datasets in the literature. Other CNN-based deep networks are already pre-trained to be used for nominal objects. In order to apply those models to our dataset, one needs to train them from scratch. Besides, our dataset has a fixed pattern, which helps its recognition and training to be faster with simple networks without dealing with complex (deep) ones. Therefore, the used model in this manuscript was compared with a CNN comprised of different numbers of layers. In each case, different combinations of hyperparameters were compared to evaluate the model performance. The metrics of sensitivity, specificity, and accuracy were chosen to compare the network performance with detailed mathematical formulas as follows:

(19) $$Sensitivity=\frac{TP}{TP+FN}$$

(20) $$Specificity=\frac{TN}{TN+FP}$$

(21) $$Accuracy=\frac{TP+TN}{TN+TP+FP+FN}$$

where TP,TN,FP, and FN represent true positive, true negative, false positive, and false negative, respectively. Figure 14 demonstrates the sensitivity and specificity analysis among the three different network layers of 1, 2, and 3. It is evident that the number of layers has a negligible impact on the model effectiveness as the measured sensor responses are simple with a fixed pattern. Therefore, the simplest model with only a single CNN layer was chosen to be used in this analysis.

The CNN model structure with the highest accuracy could be achieved as follows: a single convolutional layer with 32 filters with the size of (3,3) is followed by a max pooling layer. In order to provide features for the classifiers, filter maps were flattened. The output layer contains 11 nodes corresponding to 11 water concentration samples. Since the problem is a multi-class classification task, it requires using the softmax activation function. A dense layer with 100 nodes is between the feature extractor and the output layer to interpret the features. All layers except the output layer use ReLu as the activation function. The stochastic gradient descent optimizer with learning rate lr=0.01 and momentum β=0.9 was used to optimize the categorical cross-entropy loss (CCE) function. Here, to monitor the model performance, accuracy and loss were chosen as the evaluation metrics.

**Figure 15:**
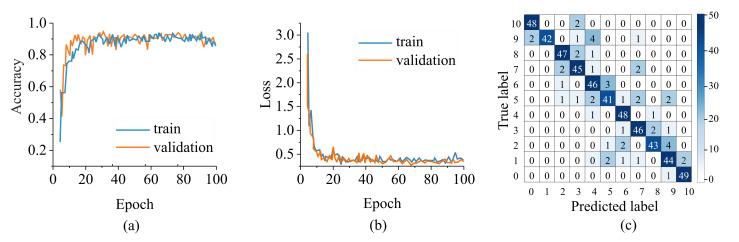
CNN Classifier (**a**) accuracy and (**b**) loss per epoch number. Confusion matrix for classifying the samples into categories of water percentage from 0→100% with 10% increments. (**c**) Confusion matrix for the selective microwave sensor.

The saved Style-GAN generator model was used to create 500 heat maps per concentration from which 80% were used as the training set and 20% as the validation set to train the CNN classifier. Once the CNN was trained, 50 unseen heat maps per class obtained from measurement were used to test the CNN classifier performance as the external dataset. Figure 15a,b depicts the classifier’s convergence curves including the accuracy and loss metrics over 100 epochs. The confusion matrix depicted in Figure 15 c shows a high accuracy of 90.7% over all classes. This confirms the selectivity of the sensor to the desired MUT (here water) regardless of the background material.

### 3.5. Model Uncertainty

**Figure 16:**
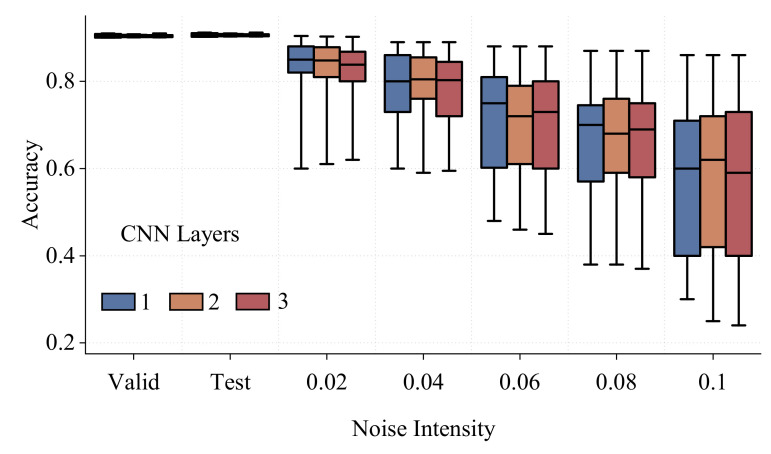
Uncertainty of the model with various noise levels being increasingly introduced with the corresponding standard deviation σ∈0.02,0.04,0.06,0.08,0.1.

Regarding the robustness of the model, high probabilities in classification do not guarantee the high certainty of the model. In the proposed work, the Gaussian process (GP) was applied to the features of the last layer of the CNN model, which allows for the practical evaluation of uncertainty bounds without losing the power of the deep learning model. The activations of the last hidden layer were extracted as the features that were used to train the GP. The GP comprises a Matern kernel [41] with ν=3/2 with white noise to account for noisy data. Several different states of white noise level were increasingly introduced to the model to distort it. As a result, the model with various noise levels according to standard deviation σ∈0.02,0.04,0.06,0.08,0.1 in the last layer accounting for the noisy data was evaluated for three different layers 1, 2, and 3, and the accuracy is reported as shown in Figure 16. The test and validation set are also included for comparison. It is clear that the single-layer CNN model has comparable performance as the ones with more layers. As a result, a single-layer CNN was used to conserve resources and simulation time.

### 3.6. Comparative Analysis

A comparative analysis is detailed in Table 2 where planar microwave sensors are examined with respect to the frequency of operation, method of operation, materials used, and proposed applications. Some sources [42] report sensors to analyze the composition of the solution using a multi-resonance approach. However, these sensors are not developed to selectively react to only a single material. In contrast, the proposed system enables the sensor to be selective only to a specific MUT in a complex matrix. For instance, in biological analysis of the body state such as the glucose level in the blood, several analytes including fructose, galactose, caffeine, etc., also undergo changes as a result of food intake. Therefore, designing a sensor to be selective to only glucose within such a complex matrix is the ultimate goal to enable sophisticated clinical applications. Hence, using the proposed sensor, the effects of single or multiple background interfering impacts can be nullified. In addition, the proposed approach introduces a novel method to increase the number of measured materials with high accuracy. Therefore, the measurement inaccuracies due to human error or setup displacements can be avoided.

On the limitation of the proposed study, the sensitivity of the microwave sensor to the placement of the MUT, as well as the surrounding medium in the sensor’s proximity are of high importance. Typically, microwave devices need to be protected from environmental electromagnetic interference for high accuracy and repetition. Lastly, the number of measurements to be considered in the training process needs to be high for better convergence; however, too many measurements usually include higher human errors. In our study, we tried to avoid such errors with the help of StyleGAN, whose performance is tied to available resources (e.g., memory, GPU, etc.).

## 4. Conclusions

In this work, a multi-resonator sensor using metamaterial transmission line zeroth-order resonators was developed that covers a wide range of frequencies from 3 GHz to 5.5 GHz. The resonator was exposed to binary mixtures of water in {methanol, ethanol, and acetone} with concentrations ranging from 0%→100% with increments of 10%. The sensor was developed to be selective to only water regardless of the host medium. In this process, a convolutional neural network was used as the classification algorithm. Sensor response ($|S_{21}|$ and $∡S_{21}$) was converted into heat maps with the phase vs. frequency diagram colored by the magnitude of $S_{21}$. These images were fed to the CNN as the input. A large number of images were generated using Style-GAN on the heat maps from two sources. Using Style-GAN, heat maps of intermediate concentrations were generated with a high PSNR of 27.4 dB. Finally, the classification of all samples into 11 categories (0%:10%:100% all-inclusive) confirmed a high accuracy of image generation by Style-GAN combined with the CNN classifier of 90.7%. This approach promises the development microwave sensors selective to a desired material of interest.

## Figures and Tables

**Table 1:** Sensor parameters’ dimensions and substrate information.

Parameter	g	*W_stub_*	*l_IDC_*	*I_stub_*_1_	*I_stub_*_2_	*I_stub_*_3_
Value [mm]	0.4	0.8	4	3	4.5	2
Rogers 5880		$\varepsilon_{substrate}$	tan(δ)	Thickness		
		2.2	0.0009	0.5		

**Table 2:** Comparison table for various sensors studying selectivity features.

Ref	Sensing Technique	$f_{\mathrm{res}}$ [GHz]	Method	Material	Applications/Metrics
[42]	Planar SRR ^1^	0.5–4.5	Multi-harmonics analysis using ANN	Water/ethanol/gasoline	Gas and oil industryMSE: 0.057%
[43]	Planar SRR	1–10	Mathematical analysis	Saline water/oil/brine	Detection of water and brine in oil—Error: 0.87%
[44]	Xethru Radar	6–7.75	Time-domain analysis	Water and fat in raw milk	Dairy industry MSE: 0.29%
[45]	μ strip SRR	0.5–2.2	logarithmic regression method	CaCl_2_, NaCl, KCl, MgCl_2_ and Na_2_CO_3_	Concentrations of salts in water—error: ±1 MHz
[46]	CSRR ^2^	4.2–8	Microwave interferometry	Ethanol/methanol/ butan-1-ol/propan-1-ol	Error: 1.1%
[47]	CBCPW	0.5–40	Stokes–Einstein–Debye equation	Water/Alanine	Biosensing and bioelectromagnetics
[48]	Multi-Slot SRR	0.1–6.5	Multi-resonance analyzed using AI	Water/methanolethanol/peroxide/acetoneglycerol/propanol	MSE: 0.13%
[4]	Single wire coil	0.2–4.5	Volumetric analysis of the mixtures	Water–clay/bitumen	Bitumen–water–clay analysis—MAE: 4%
[49]	Planar SRR	1.1, 2.2	Mathematical analysis of ε in mixture	Ethanol–water–glucose	Fermentation process in food industry—Error: 0.087%
This work	0th-order resonator	3–6	CNN-based selective classifier	Water in methanol, ethanol, acetone	Selectivity in biomedical/CCE: 0.21%accuracy: 90.7%

## Data Availability

The data are not publicly available due to data privacy restrictions.
